# Anemia

**Published:** 1844-08

**Authors:** John McLean

**Affiliations:** Professor of Materia Medica and Therapeutics in the Rush Medical College


### *Anemia.* By John McLean, Professor of Materia Medica and Therapeutics in the Rush Medical College.

*What is meant by Anemia—Its occurrence in Females during the periods of Gestation and Lactation, the subject of the present article. Symptoms—Causes.*

Anemia, in its strict acceptation, means absence or deficiency of blood; but it is frequently employed to indicate a deficiency of some of its essential constituents, especially of the coloring matter and fibrin. Thus considered, it signifies two conditions of the blood. The one is a deficiency of the whole mass of sanguine fluid, and is called complete; the other is the absence or defect of some of its essential elements or proximate principles, and is called incomplete. The Anemia to be considered in the present article, is not only a deficiency of the whole mass of blood, but a disproportionate absence of some of its component parts.

We will now proceed to the consideration of Anemia, as it occurs in females, during the term of utero-gestation; but more particularly, during the period of lactation. The disease, we shall divide into two stages.

*Symptoms of the* 1*st.*—There is a general sense of languor and exhaustion, which is much increased by exercise. The surface of the body loses its natural color, and becomes pallid. Indigestion is a troublesome symptom. There is a burning, gnawing sensation in the stomach, which at times is very distressing. The appetite is variable; at times craving, and again absent for days. When good, the patient generally indulges so freely in eating, as to bring on an aggravation of all the symptoms, and a distressing turn of diarrhœa. In all cases which I have seen, diarrhœa, more or less, has been a constant symptom. Ulceration of the tongue and inside of the mouth is so common, that, by many, the disease has received the name of nursing sore mouth. In places where this disease is prevalent, females, sometimes become very much alarmed at the occurrence of ulcers on the inside of the mouth, supposing themselves to be attacked with this formidable malady. However, there are many cases of such ulceration, which are not at all connected with anemia; but on the other hand, I do not recollect of seeing a case of anemia in the female during the period of lactation, where they were absent. In some cases, the ulcers are small and many; in others they are more large and less numerous. They are generally quite irritable and painful. When constipation of the bowels exists for some days, they are generally much worse; and on the occurrence of diarrhœa, they are better, and if quite small, sometimes totally disappear, but soon return. The pulse varies from 90 to 100 and is small. Slight exercise increases greatly the frequency of the pulse, and produces palpitation, and difficulty of breathing. The capillary circulation becomes languid, and the surface has a sickly appearance.

*2d Stage.*—The general languor and exhaustion of the first are now greatly increased. There is a disinclination to move about; and slight exercise causes a laborious and hurried breathing, which is attended with an oppressive sense of suffocation. The surface of the body now, becomes quite pale. In such, as are naturally of a light complexion, it becomes almost white. The prolabia loose their florid hue and become extremely pale. The tongue and inside of the mouth are pallid, or of a faint yellow color. The adnata of the eyes are void of vessels carrying red blood, and become almost of a perfect white. In well marked cases, the ears are extremely white, and quite translucent. This marked degree of whiteness, of the skin, does not attend all cases. In such as were formerly of a mixed and sanguine temperament, the surface assumes a sickly and sallow complexion, easily distinguished from jaundice, although, by the inexperienced it is sometimes mistaken for it. Indigestion is increased; the appetite fails; and the diarrhœa becomes more constant and watery. In many cases there is a copious secretion, from the glands in the mouth. In one case, which I attended, it amounted to about 16 oz. daily. In some cases, the ulcers remain about as they were in the first stage; in others, they become large and fotid. Sometimes but one large ulcer exists on the inside of one of the cheeks. The pulse is now quite small, feeble, and frequent, numbering about 130 a minute. The carotids throb, so as to be distinctly visible, to even the careless observer. At times, (but especially after exercise) the heart so palpitates as to become quite distressing to the patient. There is intolerance of light and sound. The respiratory functions suffer much. Even in a state of repose there is much difficulty of breathing; but when under the influence of exercise the respiration becomes laborious and hurried, and is attended with an oppressive sense of suffocation. The capillary circulation is languid and feeble. A constant disposition to œdema exists; the feet become swollen, the eyelids turged, and the face puffy; and the flesh looses its firmness and becomes soft and flabby. This state arises from the excess of the serous portion of the blood, and is strongly characteristic of *anemia.* The body emaciates, and the system, as if highly tenacious of life, feeds upon itself until all that is capable of supporting existence becomes exhausted. The appearance now becomes highly cadaverous; the eyes loose their expression; there is great restlessness; the breathing becomes short and laborious, with gaspings for breath. Insensibility slowly creeps on, and death finally closes the scene.

Good, in speaking of the extreme emaciation that takes place in amenia, and of the system feeding upon itself until the last drop of blood, capable of supporting existence, becomes exhausted, says: "The fault does not, therefore, so much seem to have been in the secernent system, or assimilating powers, as in the lacteals or digestive functions; in the commencement, rather than in the termination, of the chain."

*Exciting Causes.*—The causes of *anemia* are many and various. Among them may be enumerated the deprivation of food, or that which is void of nutritive qualities; copious and frequent venesections; long confinement in an impure atmosphere; dark and damp situations; long continued disease, more particularly if it is connected with the function of nutrition; protracted intermittents, lactation, &c.

In this town (Jackson, Mich.) anemia, within the last few years, has frequently occurred in females, and some few cases have proved fatal. I have been informed that such has been the case in many other western places. It is much more common in the western, than eastern States. Why is it? May not the same causes that operate in producing intermittents be more or less concerned in the production of this state of the system? I have observed, that those were more frequently the subjects of anemia, who lived in such places, as to render them freely under the influence of *marsh miasmata*, than others, who were not thus exposed. Every person, at all acquainted with intermittents, is well aware, how anemic that individual becomes, who has for a long time been laboring under an intermittent. And sometimes after the regular intermittent has been broken up, do we see the person languid, sickly, debilitated and anemic—and all this without any well marked symptoms of organic disease. That place, where marsh miasmata and intermittents abound, is always noted for its pale faces. Under certain conditions, marsh miasmata may operate quite injuriously upon the system, without producing well marked remittents or intermittents. May not this be the case with those *anemic* patients who never had intermittent? Many of them labor under a low grade of remittent, which escapes their own observation, and often, for a long time, that of the physician.

*(To be continued.)*

